# Therapeutics of Charcot neuroarthropathy and pharmacological mechanisms: A bone metabolism perspective

**DOI:** 10.3389/fphar.2023.1160278

**Published:** 2023-04-12

**Authors:** Liang Liu, Qiong Wang, Yan Zhang, Jingqi Liang, Peilong Liu, Hongmou Zhao

**Affiliations:** Department of Foot and Ankle Surgery, Honghui Hospital of Xi’an Jiaotong University, Xi’an, China

**Keywords:** bone metabolism, Charcot neuroarthropathy, pharmacology, RANKL, therapeutics

## Abstract

Charcot neuroarthropathy (CN) is a chronic, destructive, and painless damage of the skeletal system that affects the life quality of patients. CN, with an unclear mechanism, is characterized with invasive destruction of bones and a serious abnormality of bone metabolism. Unfortunately, development of an effective prevention and treatment strategy for CN is still a great challenge. Of note, recent studies providing an insight into the molecular mechanisms of bone metabolism and homeostasis have propelled development of novel CN therapeutic strategies. Therefore, this review aims to shed light on the pathogenesis, diagnosis, and treatment of CN. In particular, we highlight the eminent role of the osteoprotegerin (OPG)-receptor activator of nuclear factor-κB (RANK)-RANK ligand (RANKL) system in the development of CN. Furthermore, we summarize and discuss the diagnostic biomarkers of CN as well as the potential pharmacological mechanisms of current treatment regimens from the perspective of bone metabolism. We believe that this review will enhance the current state of knowledge on the diagnosis, prevention, and therapeutic efficacy of CN.

## 1 Introduction

The incidence of diabetes has steadily surged among the aging population all over the world (Ahmad et al., 2022). Chronic and uncontrolled blood glucose disorder can result in a series of harmful effects on the body. One of the most serious complications of diabetes is neuropathy, which is reported to affect up to 60% patients diagnosed with diabetes (Zakin et al., 2019). A considerable percentage of patients with symmetrical distal neuropathy is affected with a long-term and destructive disease called Charcot neuroarthropathy (CN) (Dardari, 2020). CN is a chronic, destructive, and painless damage of the skeletal system that affects the life quality of patients. While the mechanism underlying its pathogenesis is yet unclear, CN is characterized with invasive destruction of bones and a serious abnormality of bone metabolism (Pitocco et al., 2019). Unfortunately, effective prevention and treatment for CN is still a great challenge.

**FIGURE 1:**
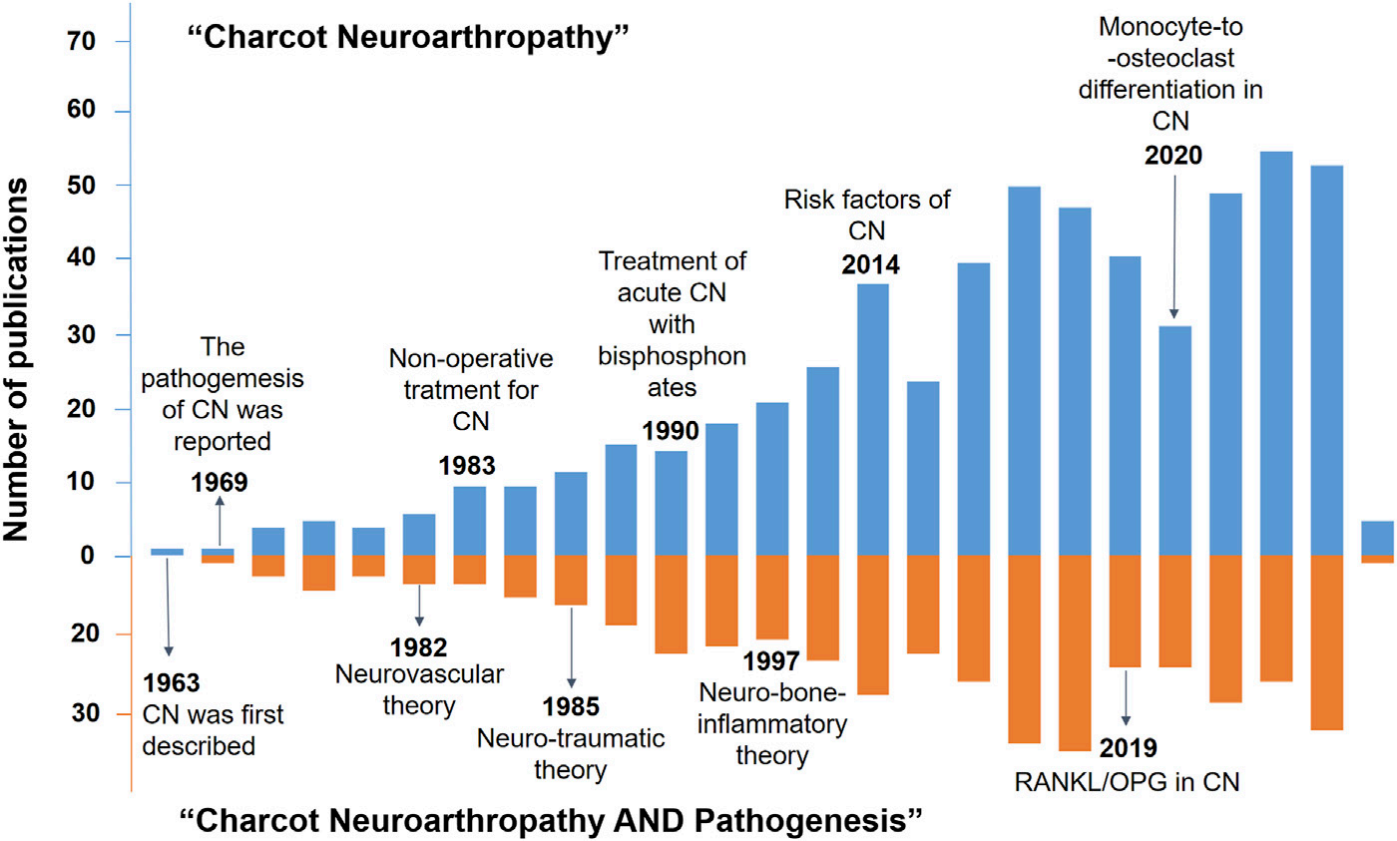
Timeline of the key development events in the knowledge (blue histogram) and pathogenesis of CN (orange histogram).

CN was first described in a Spanish article by Temesio et al. (1963), while its pathogenesis was first explained by (Bruckner and Kendall, 1969). Over the past couple of decades, several research studies have explored the potential molecular mechanism of CN (Dodd and Daniels, 2018; Botek et al., 2019). To our knowledge, the definitive mechanisms of CN are elusive, and there are several representative theories related to CN pathogenesis (Shapiro et al., 1998). Given the crucial role of the osteoprotegerin (OPG)-receptor activator of nuclear factor-κB (RANK)-RANK ligand (RANKL) system in the development of CN (Bruhn-Olszewska et al., 2017), bone metabolism was considered to be actively involved in the pathological alteration of CN. Since then, numerous studies have been conducted to understand CN and its pathogenesis, as exemplified in Figure 1.

The studies published so far have improved our understanding of CN pathogenesis as well as the progress in its diagnosis and treatment. In fact, recent advances in the knowledge of the molecular mechanisms of bone metabolism and homeostasis have provided a promising direction for scheduling new CN therapeutic strategies. However, the underlying mechanisms and the actual efficacy of current therapeutic strategies for CN are yet to be elucidated. During the novelty retrieval via Web of Science search engine, we set “Charcot Neuroarthropathy” and “Charcot Neuroarthropathy AND Pathogenesis AND Bone metabolism” as search topics, which led to a dramatic decrease in the number of publications from 650 to 28 (Table 1). Thus, the correlation between CN pathogenesis and bone metabolism has not been extensively investigated.

**TABLE 1:** The comparison of different search topics.

Search	Topic	Items found	Date
#4	Charcot Neuroarthropathy AND Pathogenesis AND Bone metabolism	28	2023.01.28
#3	Charcot Neuroarthropathy AND Pathogenesis AND Metabolism	46	2023.01.28
#2	Charcot Neuroarthropathy AND Pathogenesis	330	2023.01.28
#1	Charcot Neuroarthropathy	650	2023.01.28

Recently, several comprehensive reviews have summarized and discussed the excellent stride of bone metabolism in CN development (Bruhn-Olszewska et al., 2012; Yates et al., 2020). However, it remains a lack of comprehensive summarization of BN’s pathogenesis specially with a bone metabolism perspective. Considering this issue and the potential involvement of bone metabolism in the development of CN, we believe that it is necessary and timely to present a review on this topic to shed light on the pathogenesis, diagnosis, and treatment of CN. Herein, we highlight the eminent role of the OPG-RANKL-RANK system in the development of CN. Furthermore, we summarize and discuss the diagnostic biomarkers of CN as well as the potential pharmacological mechanism action of current treatment regimens from the prospective of bone metabolism. We believe that this review will improve the current state of knowledge on the diagnosis, prevention, and treatment of CN.

## 2 Pathophysiology of CN

**FIGURE 2:**
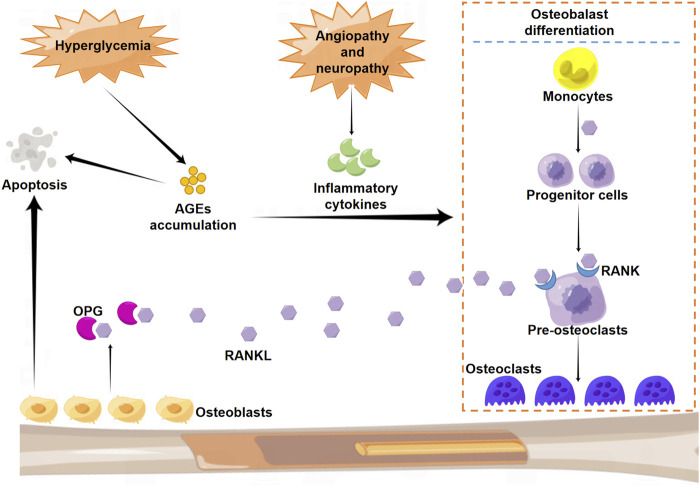
Schematic illustration of the molecular mechanism of osteoclast differentiation. RANK: nuclear factor-κB; RANKL: RANK ligand.

While numerous studies in the past decades have focused on the pathogenesis of CN, the definitive molecular mechanism remains unknown. Increasing evidence indicates multifactorial events that contribute to this pathogenic condition, among which diabetic neuropathy and angiopathy, mechanical stress and injuries, excessive and persistent inflammation, and disturbance in bone metabolism are thought to play predominant roles (Trieb, 2016). In particular, the impairment of proprioception caused by peripheral neuropathy is believed to aggravate joint instability and, therefore, markedly increase the risk of injury from the small mechanical stress (Botek et al., 2019). The consequent transformation in the physical biomechanics of the foot is known to induce abnormal plantar pressure and add to subsequent localized injuries (Botek et al., 2019). Under physiological conditions, there is a relative balance in bone metabolism maintained by appropriate regulation between osteoclasts that drive bone resorption and osteoblasts responsible for bone formation. However, overexpression and secretion of inflammatory cytokines, including interleukin (IL)-1β and IL-6, under CN pathogenic condition induce the over-activation of the RANKL-RANK system and consequently impair the balance of bone metabolism. This phenomenon can lead to excessive bone resorption and joint destruction (Lee et al., 2003). Osteoclast activation is initiated by an increase in RANKL level in the OPG- RANK-RANKL system. Under diabetic condition, hyperglycemia enhances the production and accumulation of advanced glycation end products (AGEs), which induce bone resorption by promoting osteoblast apoptosis and osteoclast differentiation (Pitocco et al.,2019). In addition, micro-trauma and fracture perpetuate inflammation *via* increased expression of cytokines and further aggravate bone resorption (Figure 2).

## 3 Regulatory factors in the development of CN

### 3.1 OPG-RANKL-RANK system associated with bone metabolism

The dysregulation in the balance between inflammatory and anti-inflammatory activities is highly associated with the development of CN. As a classical signaling pathway involved in the regulation of bone metabolism, the OPG-RANK-RANKL system can be significantly upregulated by the excessive release of inflammatory cytokines (Hou et al., 2012). In the bone matrix, RANKL is associated with differentiation and maturation of osteoclast precursor cells (Yang et al., 2020). In general, mature osteoclasts and several stem cells express RANK receptors that bind to RANKL and consequently exert multiple biological functions (Tyrovola, 2017). The activation of the signal cascade following binding between RANK and RANKL leads to the recruitment of tumor necrosis factor receptor-associated factors (TRAFs) to the cytoplasmic domain of RANK and the consequent maturation of preosteoclasts (Ma et al., 2012). Of note, OPG serves as a soluble decoy receptor for RANKL and ameliorates bone absorption by blocking the binding of RANKL to RANK (Wang et al., 2022). Although the crosstalk between RANKL and OPG drives bone metabolism, other factors such as AGEs, reactive oxygen species (ROS), and inflammatory cytokines are also involved in the regulation of this process and contribute to the progression of CN. For instance, a prior study reported that inflammation can be aggravated by the accumulation of AGEs, and the persistent if a crucial stimulation for the development of CN (Hockenbury et al., 2007).

### 3.2 The role of the OPG-RANKL-RANK system in CN development

Any dysfunction in the OPG/RANKL/RANK system can result in unexpected bone absorption and a series of events, including CN (Ndip et al., 2011). In a pioneering study, Alexander group reported that the activation of the OPG/RANKL/RANK system can aggravate the development of CN by mediating arterial calcification. This study provided a promising direction for anti-CN therapies through targeting of the OPG/RANKL/RANK system (Ndip et al., 2011). Similarly, Shanahan et al. reported inflammatory cytokine accumulation and *RANKL* gene expression in the area of bone destruction among CN patients, and highlighted the close correlation between the OPG/RANKL/RANK system and CN pathogenesis (Petrova and Shanahan, 2014).

Several studies have demonstrated the critical involvement of OPG and RANKL variants in bone destruction observed among CN patients (Roshandel et al., 2010; Wang et al., 2012). For instance, *OPG* gene single nucleotide polymorphism (SNP) was found to be highly involved in the development of diabetic CN. In particular, there was a positive correlation with G alleles for both the OPG 1181G>C and 245T>G variants in CN patients as compared to patients with diabetic neuropathy and healthy controls (Pitocco et al., 2009). Clinical data revealed much higher RANKL and OPG protein levels in the serum of CN patients than in healthy controls, consistent with a high RANKL/OPG ratio among CN patients (Jansen et al., 2018). Evidence suggests that elevated serum levels of RANKL in CN patients can enhance osteogenic differentiation and mineralization of vascular smooth muscle cells (Ndip et al., 2011), which can eventually aggravate the ischemic necrotic lesion of the limb. Together, all these findings reveal the dominant role of the OPG-RANKL-RANK system in the development of CN pathogenesis.

### 3.3 Neuro-bone-inflammatory axis in CN

While the molecular mechanism underlying CN pathogenesis remains elusive, studies have speculated a critical role of the neuro-bone-inflammatory axis in the pathogenic development of CN (O'Connor et al., 1985). CN patients were found to have lower bone density in the affected limbs than other neuropathic participants (Young et al., 1995). In particular, previous studies on bone metabolism revealed enhanced osteoclastic activity, instead of osteoblastic activity, in acute and chronic CN patients (Jeffcoate, 2005). Furthermore, inflammatory cytokines such as IL-1β and IL-6 are found to be highly involved in the pathogenesis of CN through induction of nuclear factor kappa B (NF-κB) and RANKL activities (Connors et al., 2018). Although some studies reported that there was no significant difference in the ratio of RANKL/OPG between CN patients and participants without CN (Connors et al., 2018), *in vitro* and *in vivo* results have proposed the importance of this system in the development of CN (Geusens et al., 2006). Together, these studies suggest a “neuro-bone-inflammatory theory” in CN that might explain the correlation between the intercellular communication of neural cells and bone cells as well as the crucial role of inflammatory signaling in CN regulation.

## 4 Pharmacological therapies of CN

### 4.1 Therapeutic agents targeting bone metabolism

The typical radiographic characteristics of Charcot joint involve excessive bone resorption and joint destruction, which indicate the imbalance in bone metabolism. A wide array of studies have shown abnormal upregulation in the expression of multiple bone resorptive markers among CN patients (Yates, et al., 2020). It is well-documented that therapeutic strategies targeting inhibition of excessive bone absorption can be promising for CN therapy (Schulze et al., 2022). The most widely used treatment is the application of bisphosphonates (BPs), which were first synthesized in 1865 and have been used in medicine since 1968. BPs exhibit a robust anti-resorptive activity and alleviate bone turnover (Jude et al., 2001).

Mechanistically, BPs have high affinity for bone minerals and can be deposited in the mineralized bone matrix, wherein they exert pharmacological functions before bone resorption (Dissanayake et al., 2012). Their high affinity for bone minerals and selective uptake by osteoclasts under the resorptive condition ensures specific toxicity only to osteoclasts. Non-nitrogen-containing BPs are first-generation agents that function as cytotoxic adenosine triphosphate analogues in osteoclasts. The deposition of these toxic adenosine triphosphate analogues results in the apoptosis of osteoclasts (Rastogi et al., 2021). Second- and third-generation BPs such as alendronate, ibandronate, and zoledronate have a nitrogen side chain bound to the central carbon that enhances their potency. Mechanistically, upon internalization, nitrogen-containing BPs suppress the activity of farnesyl pyrophosphate (FPP) synthase, which is responsible for production of cholesterol and isoprenoid lipids (Kavanagh et al., 2006). This phenomenon leads to suppression of isoprenylation of guanosine triphosphate-binding proteins such as Ras, Rho, and Rac, which are closely associated with cell proliferation, maturation, and differentiation (Li et al., 2021). The suppression of Ras signaling pathway in osteoclasts leads to defective intracellular vesicle transportation and failure of ruffled border formation, which results in amelioration of bone resorption (Cremers et al., 2019). In addition, FPP synthase inhibition can also prevent bone resorption through induction of osteoclast apoptosis (Naylor et al., 2016).

### 4.2 Anti-inflammatory therapies

The pathogenic characteristics such as excessive and persistent inflammation play a significant role in the development of CN. Emerging findings have uncovered the roles of several signaling pathways, wherein excessive inflammation is known to contribute to osteoclast over-activation in CN. This observation can provide clues for development of promising therapeutic options for CN (Sinacore et al., 2017).

A variety of cytokines and hormones are involved in regulation of OPG/RANK, and can thereby mediate osteoclast maturation and differentiation (Zaidi et al., 2003). Stimulants of osteoclastogenesis, including IL-1, IL-6, tumor necrosis factor (TNF)-alpha, parathyroid hormone, and calcitonin, play an eminent role in osteoclast function and bone resorption. For example, a clinical randomized control study suggested that teriparatide (recombinant human parathyroid hormone) can enhance bone remodeling in CN patients by mediating an osteoanabolic effect and can increase the mineral density of foot bones (Rastogi et al., 2019). Similarly, indicated that denosumab could provide a beneficial effect on prevention of bone and joint destruction, together with a metabolic effect in CN treatment (Carves et al., 2021). Evidence suggests a link between the inflammatory responses and bone metabolism abnormalities in CN (Pitocco et al., 2008). In particular, excessive inflammation can be initiated by fracture or other micro-trauma issues, which lead to production and release of several proinflammatory cytokines that can stimulate RANKL overexpression and osteoclast maturation. Abnormally upregulated TNF-α, IL-1, and IL-6 levels have been demonstrated as critical prognostic markers for CN (Baumhauer et al., 2006).

Therefore, it was assumed that TNF-α inhibitors and high-dose corticosteroids (that suppress NF-κB expression) could serve as beneficial agents for the treatment of CN. However, clinical data and verification of efficacy of these agents are highly desired. In addition, certain anti-inflammatory therapies, including inhibitors of RANKL, NF-κB, and IL-1β, have already been applied in animal experiments to ameliorate inflammation in arthritis (Molines et al., 2010).

### 4.3 Nutritional and pharmacological agents

The interaction between diabetes, neuropathy, and excessive inflammatory response is one of the main causes for CN development that can lead to bone absorption and joint deformity (Rader and Ruter, 2022). Oxidative stress (OS) can initiate activation of inflammation and eventually lead to bone destruction (Rizzo et al., 2017). AGEs are over produced in response to OS and can mediate apoptosis of osteoblasts (Rizzo et al., 2017). The receptor binding to AGEs (RAGE) is known to be closely associated with the pathogenesis of CN (Korzon-Burakowska et al., 2012). RAGE was previously shown to increase the activity of RANKL and contribute to osteoclastogenesis of the bone (Yaturu, 2009). Furthermore, RAGE expression was found to be upregulated in CN and was associated with the development of atherosclerotic lesions and vascular calcification through an increase in the expression of bone morphogenetic protein 4 in arteries (Witzke et al., 2011).

Diabetes is associated with an increase rate of lipoperoxidation, while vascular calcification (VC) is linked with upregulation in oxidized low-density lipoprotein expression (Thomsen et al., 2010). In a prior study, antioxidants such as 4-hydroxy-tempol, alpha-lipoic acid, and apocynin were shown to prevent calcification in the femoral artery, but only apocynin significantly alleviated femoral artery calcification (Brodeur et al., 2014). Furthermore, alpha-lipoic acid was found to markedly decrease aortic calcification in diabetic mice by inhibiting apoptosis of endothelial cells and restoring the mitochondrial function (Kim et al., 2012). As a commonly used nutritional agent, vitamin D has been demonstrated to protect pancreatic beta cells from OS through activation of endogenous antioxidant pathways (Wei et al., 2018). Taken together, nutritional and pharmacological agents can be used as potential therapeutic options for CN treatment.

## 5 Challenges and perspectives

Considering the tremendous surge in its incidence and prevalence, diabetes has become the most common cause of CN affecting the foot and ankle. From the perspective of bone metabolism, the main goal of pharmacological intervention involves suppression of excessive inflammation and bone resorption. Therapeutic strategies targeting inhibition of osteoclast function and pro-inflammatory signaling can provide a promising direction for CN treatment. Anti-resorptive treatments, especially with BPs, have been used in animal models and randomized clinical trials. Although there is no evident verification of an ideal dosage regime and true long-term validity, clinical trial results suggest improved symptom control, a more rapid decrease in the foot temperature, and a marked decline in bone resorption marker levels without any serious side-effects. While evidence suggests the direct involvement of monocytes in the pathogenesis of CN, further research focused on the monocyte-to-osteoclast differentiation process may give us a better insight into early prevention strategies for this pathogenic condition. Early diagnosis is still the best strategies for the management of patients with CN. In patients with diabetes and lower extremity neuropathy, any delicate injury deserves necessary observation due to the tendency of the limb to proceed to a Charcot’s process (Galhoum et al., 2021). In summary, the accumulation of knowledge about the molecular pathways underlying the pathogenesis of bone metabolism in CN is of great importance to facilitate advances in pharmacological treatments.
